# Spontaneous Abortion and Myocardial Infarction: A Mendelian Randomization Investigation and Transcriptomic Analysis

**DOI:** 10.5334/gh.1392

**Published:** 2025-02-06

**Authors:** Shiqing Xiang, Qingxia You, Fangxiang Mu, Nian Zhang

**Affiliations:** 1 Department of Laboratory Diagnosis, Southwest Hospital, Chongqing 400038, China; 2 Department of Reproductive Medicine, Lanzhou University Second Hospital, Lanzhou 730030, China; 3 Department of Traditional Chinese Medicine, Xinqiao Hospital, Chongqing 400037, China

**Keywords:** Mendelian randomization, myocardial infarction, spontaneous abortion, causal relationship, transcriptome analysis

## Abstract

**Background:**

A link has been found between spontaneous abortion (SA) and myocardial infarction (MI). However, there is still a lack of comprehensive knowledge regarding the genetic links and biological mechanisms between SA and MI. An investigation of the causal association between SA and MI, along with the associated signaling networks, was conducted using univariate Mendelian randomization (MR) and transcriptome analysis.

**Methods:**

Data from genome-wide association studies (GWAS) for SA and MI were analyzed using the FinnGen consortium database. To assess the causality between SA and MI, various methods were employed including inverse-variance-weighted (IVW), weighted median, simple mode, and weighted mode analyses. Sensitivity analysis was conducted using heterogeneity, pleiotropy, and the Leave-One-Out (LOO) approach. Transcriptomic analysis of the GSE60993 dataset was performed to identify differentially expressed genes (DEGs) associated with single nucleotide polymorphisms (SNPs). Following this, two bioinformatics analyses were carried out.

**Results:**

Based on IVW results, SA was found to be causally associated with MI (OR = 1.095, 95%CI 1.012–1.186). Sensitivity analysis was subsequently conducted to validate the robustness of our findings. Through differential analysis, three key genes – GNAQ, ELP3, and TES – were identified as closely linked to processes related to ribosome biogenesis, DNA replication, and congenital immune deficiency. Furthermore, strong correlations were observed with various immunologic gene sets, including the Major Histocompatibility Complex (MHC), immunoactivators, and immunosuppressors.

**Conclusion:**

This study reveals a robust causal relationship between SA and MI, highlighting genetic and immunological pathways that could inform future research and therapeutic approaches.

## Introduction

Spontaneous abortion (SA) is characterized by the termination of a pregnancy before fetal viability, generally occurring before 20 weeks of gestation (1). This condition impacts approximately 10–15% of clinically recognized pregnancies; however, the true incidence may be higher when accounting for early pregnancy losses that remain undetected (1,2). SA can exert emotional and physical effects on individuals who experience it (3,4). In addition to these immediate consequences, SA may also be associated with long-term health implications, including an increased risk of cardiovascular diseases (5).

Studies have indicated that women with a history of SA have a higher risk of myocardial infarction (MI) later in life (6,7). For instance, a nationwide cohort study found that women with a history of SA had a 1.13-fold increased risk of MI compared to women without such a history, with the risk increasing by 9% with the number of SA events (6). Another study showed that women with one, two, or three or more pregnancy losses had adjusted hazard ratios for MI of 1.1, 1.3, and 1.4, respectively, compared to those with no pregnancy loss (7). However, these studies are predominantly observational and encompass only a limited segment of the population. Numerous factors contribute to miscarriage, and when investigating causal relationships, various confounding variables can significantly affect the outcomes. To the best of our knowledge, there is no credible evidence to suggest that SA directly causes MI. Furthermore, the underlying mechanisms linking SA to MI are not yet fully understood. Understanding the underlying genetic links and biological mechanisms between miscarriage and MI is crucial for developing effective prevention and treatment strategies.

SA and MI may share a common gene, although this relationship has not been definitively elucidated. To investigate the potential shared genetic basis between SA and MI, we employed Mendelian randomization (MR) analysis. SA was used as the exposure variable, single nucleotide polymorphisms (SNPs) as instrumental variables (IVs), and MI as the outcome variable. This approach allowed us to estimate the causal relationship between SA and MI from a genetic perspective. Additionally, we performed transcriptomic analysis to identify differentially expressed genes (DEGs) and explore associated immune pathology profiles.

## Materials and methods

### Data acquisition

We obtained genome-wide association study (GWAS) data on SA (GWASID: R11_O15_ABORT_SPONTAN) and MI (GWASID: R11_I9_MI_STRICT) from the Finnish database (Table S1). Additionally, we obtained 17 MI samples (10 non-ST elevation MI and 7 ST-elevation MI cases) and seven healthy control blood samples from the GSE60993 dataset, which is accessible through the Gene Expression Omnibus (https://www.ncbi.nlm.nih.gov/geo/).

Since the publicly available data were previously cleared by appropriate ethics committees and all participants, no additional ethical approval or consent was required for this study.

### Data pre-processing

We utilized the R programming language to import the local GWAS dataset and applied a p-value threshold of 5 × 10–^6^ to filter the exposed SNPs. Subsequently, for linkage disequilibrium analysis, we aggregated the independent tools by clustering with parameters set to r² = 0.001 and a distance of 100,000 kb. Finally, the LDlink tool was employed to exclude IVs that exhibited a high correlation with the outcome, thereby minimizing potential confounding effects.

### MR univariable and sensitivity analyses

After filtering IVs, we aligned effect alleles and sizes using the Harmonise_data function from the TwoSampleMR package. We employed various MR methods, including MR-Egger, weighted median, simple mode, weighted mode, and inverse variance weighted (IVW) regression. To verify robustness of the results, we assessed heterogeneity with the mr_heterogeneity function, checked for pleiotropy using the mr_pleiotropy_test function and the MRPRESSO package, and conducted leave-one-out (LOO) analysis to ensure no single SNP influenced the overall estimate. These analyses were performed separately for the exposure factors and MI.

### DEG analysis

DEG analysis was conducted using gene expression profiles from the GSE60993 dataset, alongside the nearest genes corresponding to SNPs in the R11_O15_ABORT_SPONTAN dataset. Subsequently, the differential gene expression between MI and control samples was evaluated, with the results illustrated through box plots (*p <* 0.05). In the ensuing analysis, DEGs were identified as the primary genes of interest.

### Gene set enrichment analysis

Utilizing the ‘clusterProfiler’ package within the framework of Gene Set Enrichment Analysis (GSEA), potential Kyoto Encyclopedia of Genes and Genomes (KEGG) pathways associated with the primary genes were systematically investigated. The results were considered statistically significant at a p-value threshold of less than 0.05.

### Immune invasion analysis

We employed the single-sample GSEA (ssGSEA) algorithm, a component of the GSVA package, to calculate the relative abundance profiles of 28 distinct immune cell types. Additionally, we determined 13 immune infiltration-related pathway scores within the context of the GSE60993 dataset. The Wilcoxon test was employed to evaluate the differences in immune cell infiltration and related networks between MI samples and healthy control samples. Subsequently, Spearman correlation analysis was conducted to assess the relationship between the primary genes and the significantly different immune infiltration cells and pathways.

### Immune profile analysis

Data on immunopathological characteristics were extracted from the TISIDB database (http://cis.hku.hk/TISIDB/index.php), encompassing major histocompatibility complex (MHC) molecules, immunostimulators, and immunoinhibitors. Following this, Spearman correlation analysis was conducted on the genomes of key genes associated with distinct immunological characteristics.

### Statistical analysis

Statistical significance was established when the adjusted *p*-value was less than 0.05. Differences between cohorts were evaluated using the Wilcoxon test.

## Results

### Relationship between SA and MI

**Figure 1:**
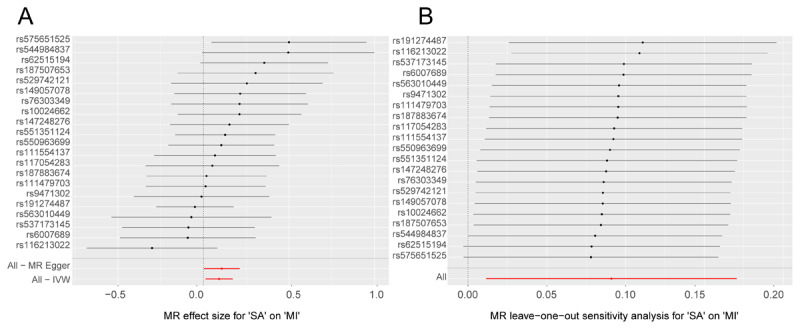
Causal effects for SA on the risk of MI by MR analyses. **(A)** Forest plots of MR analyses. IVW method demonstrated a causal association between SA and MI. **(B)** MR leave–one–out sensitivity analysis for SA on MI. LOO analysis revealed that the results were similar to IVW analysis, suggesting strong data reliability.

After rigorous screening, 21 SNPs were identified as instrumental variables. Utilizing the IVW method, we established a causal relationship between SA and MI, with an odds ratio (OR) of 1.095, a 95% confidence interval (CI) of 1.012 to 1.186, and a *p*-value of 0.025 (Table 1). The results of five algorithms were highly consistent with previous results. Therefore, SA was identified as a risk factor for MI. To evaluate the diagnostic efficiency of individual SNP loci on patient outcomes, we generated a forest plot. SNP points on the left are shown as protective factors, while those on the right are shown as risk factors. Consistent with previous results, this study demonstrated that SA is a significant risk factor for MI (Figure 1A). Finally, based on the assessment of randomness, the above results are unlikely to be influenced by potential biases there is no heterogeneity, and the results are highly consistent with the random grouping of Mendel’s second law (Table 2).

**Table 1:** The results of Mendelian randomization analyses.


EXPOSURE	METHOD	N SNP	β	SE	OR (95%CI)	*p*-VAL

SA	MR Egger	21	0.107	0.053	1.112 (1.002,1.235)	0.060

SA	Weighted median	21	0.068	0.056	1.070 (0.958,1.195)	0.231

SA	IVW	21	0.091	0.041	1.095 (1.012,1.186)	0.025

SA	Simple mode	21	0.032	0.100	1.032 (0.849,1.255)	0.755

SA	Weighted mode	21	0.029	0.088	1.029 (0.866,1.224)	0.746


SA, spontaneous abortion; IVW, inverse variance weighted model; SNP, single nucleotide polymorphisms; SE, standard error.

**Table 2:** The results of heterogeneity and pleiotropy test.


EXPOSURE	OUTCOME	METHOD	HETEROGENEITY TEST			PLEIOTROPY TEST		
	
			COCHRAN’S Q	Q_df	Q_*p* VAL	EGGER_INTERCEPT	SE	*p* VAL

SA	MI	MR Egger	18.348	19	0.499	–0.004	0.0089	0.656

SA	MI	IVW	18.553	20	0.551			


SA, spontaneous abortion; MI, myocardial infarction; IVM, inverse variance weighted model; SE, standard error.

### Sensitivity analysis

The *p*-values for the heterogeneity and pleiotropy tests exceeded 0.05, suggesting the absence of detectable heterogeneity and horizontal pleiotropy among the SNPs (Table 2). Furthermore, the outcomes of the LOO analysis were consistent with those of the IVW analysis, thereby reinforcing the robustness of the data (Figure 1B). Collectively, these findings suggest that SA exerts a significant promoting effect on MI.

### Identification and functional enrichment analysis of major MI-related genes

**Figure 2:**
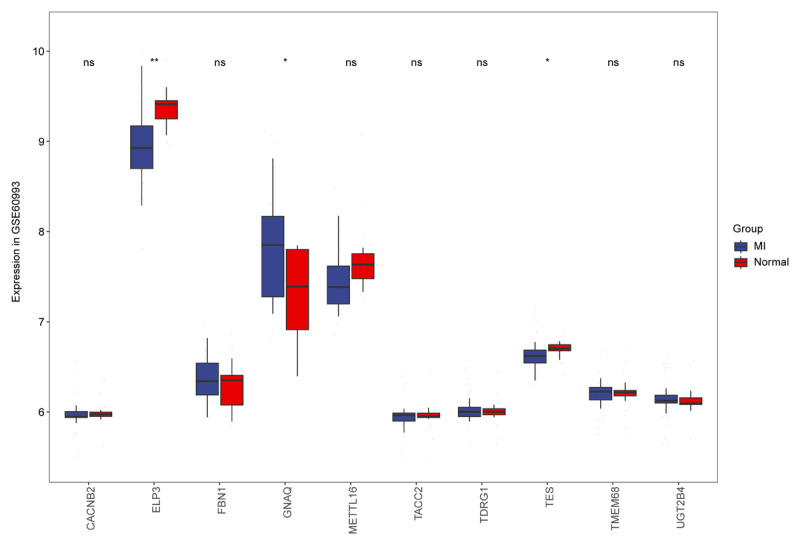
Expression levels of the genes associated with SNPs between MI and health control groups. 3 DEGs were identified between MI and healthy controls.

**Figure 3:**
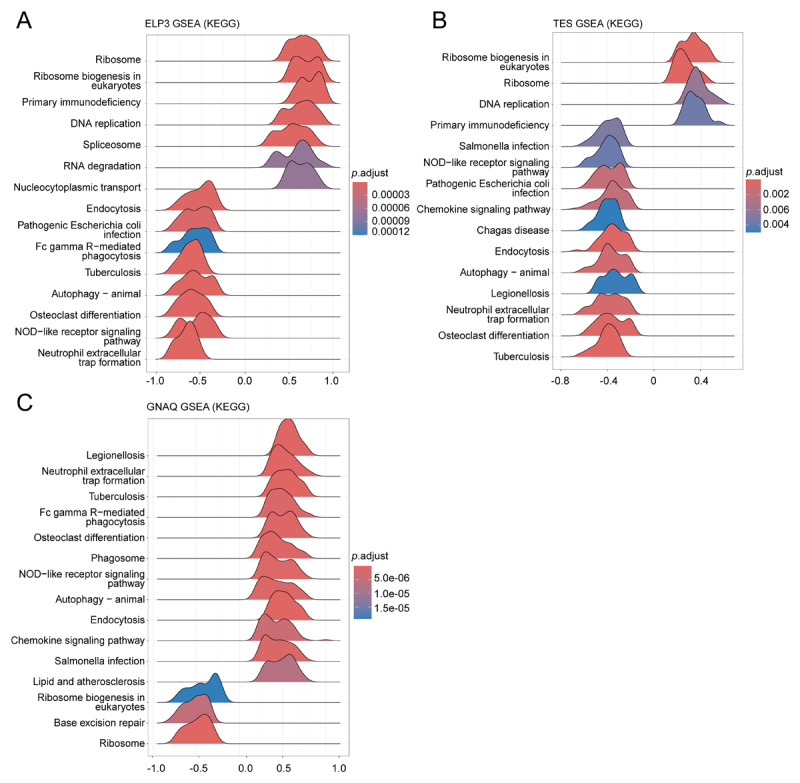
KEGG-ssGSEA results of GNAQ, ELP3, and TES gene.

In the GSE60993 dataset, we identified 10 SNP-associated genes. Among these, three DEGs were observed between MI patients and healthy controls. Specifically, GNAQ was highly expressed in MI, whereas ELP3 and TES showed low expression in MI (Figure 2). To further understand the physiological roles of these DEGs in MI, we conducted single-gene GSEA. The KEGG analysis results indicated that these DEGs are significantly enriched in pathways related to ribosome biogenesis, DNA replication, and innate immune deficiency, highlighting their critical involvement in the pathogenesis of MI (Figure 3).

### Assessing DEG function in the MI immune microenvironment (IME)

**Figure 4:**
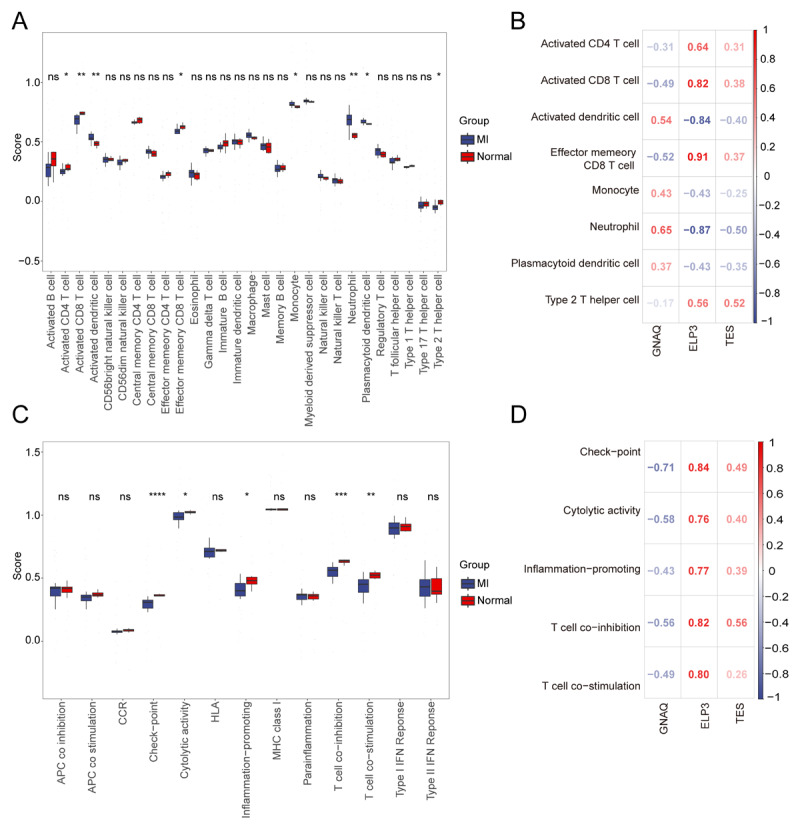
Analysis of the role of key genes in MI immune microenvironment. **(A)** The expression abundance of 28 types of immune cells in MI and health control groups. **(B)** The heatmap of the correlation between the key genes and immune cells. **(C)** The scores of 13 immune pathways in MI and health control groups. **(D)** The heatmap of the correlation between the key genes and 13 immune pathways. *: *p* < 0.05, **: *p* < 0.01, ns: not significant.

We examined the IME of MI patients by analyzing the expression profiles of 28 immune-related cell types. Our study found significant differences in the abundance of 8 immune cell types between MI patients and healthy controls (Figure 4A). In MI samples, the levels of adaptive immune cells, including activated CD4 T cells, activated CD8 T cells, and effector memory CD8 T cells, were significantly reduced compared to controls (Figure 4B). In contrast, inflammatory and myeloid cells, such as neutrophils and monocytes, were significantly increased.

Further analysis revealed a significant association between key genes and various immune cell types. Notably, ELP3 showed a strong positive correlation with both effector memory CD8 T cells and activated CD8 T cells, while exhibiting a strong negative correlation with activated dendritic cells and neutrophils. Additionally, we observed substantial differences in the scores of five immune-related networks: checkpoint, cytolytic activity, pro-inflammation, T cell co-inhibition, and T cell co-stimulation (Figure 4C). ELP3 and TES were positively correlated with checkpoint, cytolytic activity, pro-inflammatory responses, T cell co-inhibition, and T cell co-stimulation, whereas GNAQ demonstrated a negative correlation (Figure 4D).

### DEGs were intricately linked to the immunopathological profiles

**Figure 5:**
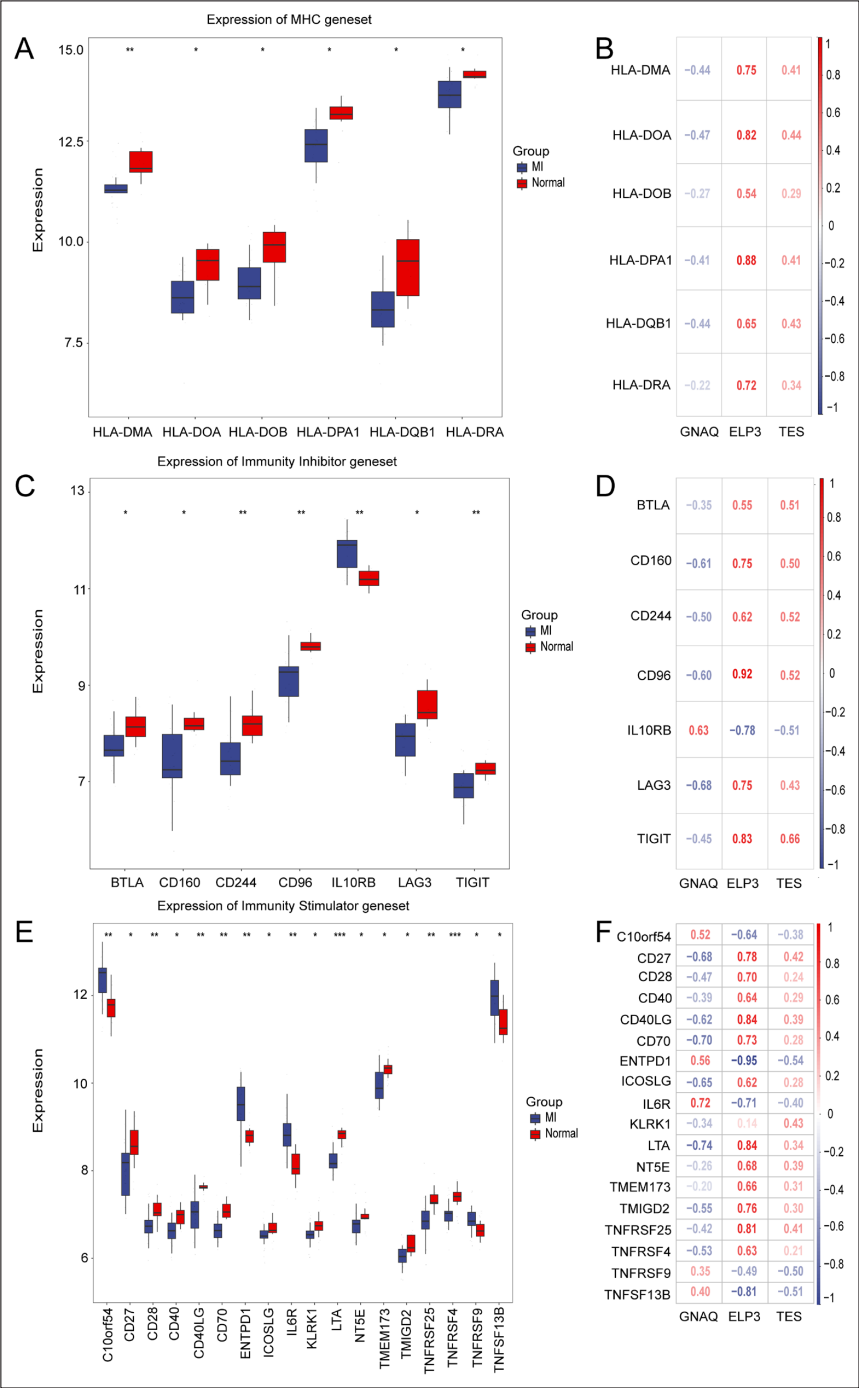
The immune characteristics associated with the key genes. **(A)** The expression levels of MHC genes in MI and health control groups. **(B)** The heatmap of the correlation between the key genes and MHC genes. **(C)** The expression levels of immunity inhibitors genes in MI and health control groups. **(D)** The heatmap of the correlation between the key genes and the immunity inhibitors. **(E)** The expression levels of immunoactivator genes in MI and health control groups. **(F)** The heatmap of the correlation between the key genes and the immunoactivator.

In the context of the MHC gene set, our findings indicate that HLA-DMA, HLA-DOA, HLA-DOB, HLA-DPA1, HLA-DQB1, and HLA-DRA were significantly downregulated in the MI group (Figure 5A). Subsequent analysis revealed that these genes exhibited a negative correlation with GNAQ and a positive correlation with ELP3 and TES (Figure 5B). Regarding immune inhibitors, BTLA, CD160, CD224, CD96, LAG3, and TIGIT were markedly downregulated, whereas IL10RB was significantly upregulated in MI samples (Figure 5C). The analysis revealed that GNAQ exhibited a negative correlation with the downregulated immune inhibitors and a positive correlation with the upregulated immune inhibitors. In contrast, ELP3 and TES demonstrated positive correlations with the downregulated immune inhibitors and negative correlations with the upregulated immune inhibitors. Notably, ELP3 showed the highest correlation with CD96 (0.92), followed by TIGIT (0.83) (Figure 5D). In the context of immune activators, the expression levels of CD27, CD28, CD40, CD40 LG, CD70, ICOSLG, KLRK1, LTA, NT5E, TMEM173, TMIGD2, TNFRSF25, and TNFRSF4 were significantly downregulated in MI samples, whereas C10orf54, ENTPD1, IL6R, TNFRSF9, and TNFSF13B were significantly upregulated (Figure 5E). Among the correlations, GNAQ exhibited the highest negative correlation with LTA (r = –0.74); ELP3 demonstrated strong correlations with ENTPD1 (r = –0.95), CD40 LG (r = 0.84), and LTA (r = 0.84); and TES showed the highest correlation with ENTPD1 (r = –0.54) (Figure 5F).

## Discussion

Numerous studies have established an association between SA and subsequent cardiovascular diseases in women, with the majority of research demonstrating a significant positive correlation between these variables. The incidence of cardiovascular disease increases with the frequency of SA, and this association is particularly pronounced in younger women (8,9). Observational studies have demonstrated that women with a history of SA are at an elevated risk of MI. In this research, we employed advanced large-scale data analysis techniques to explore the shared genetic factors between SA and MI, thereby confirming a significant positive causal relationship between SA and MI risk. These findings enhance our understanding of the role of SA in the etiology of MI and hold substantial clinical significance.

MI is precipitated by coronary atherosclerosis, which leads to thrombus formation and consequent ischemic myocardial necrosis (10). Atherosclerosis is characterized as a chronic inflammatory condition. The pathogenesis begins when low-density lipoprotein (LDL) particles infiltrate the arterial wall and undergo oxidation, subsequently attracting immune cells, including macrophages and T cells, to the site, thereby initiating an inflammatory response. The macrophages internalize the oxidized LDL, transforming into foam cells that constitute the core of atherosclerotic plaques (11). This inflammatory milieu within the arterial wall further recruits immune cells such as neutrophils and monocytes, which secrete a variety of cytokines and enzymes, contributing to plaque instability and potential rupture (12). When plaques rupture, the exposed subendothelial components rapidly trigger platelet aggregation and coagulation, forming a thrombus that obstructs the coronary artery (13). Chronic inflammation and immune response not only promote the formation and development of plaques but also affect their stability, making them more prone to rupture. Therefore, the immune system and inflammatory response play crucial roles in the formation, development, and rupture of atherosclerotic plaques. Endothelial dysfunction resulting from systemic inflammatory processes is a common and plausible underlying mechanism of SA, particularly late and recurrent miscarriages. The resulting vascular lesions are expected to lead to poor placental development during pregnancy, resulting in SA and increasing the likelihood of MI, cerebral infarction, and renovascular hypertension (6,14).

The present study revealed substantial disparities in the prevalence of eight distinct immune cell types between MI patients and the control group. Notably, adaptive immune response cells, including activated CD4 T cells, activated CD8 T cells, and effector memory CD8 T cells, exhibited a significant reduction. Conversely, inflammatory and myeloid cells, such as neutrophils and monocytes, demonstrated a significant augmentation. Monocytes play an important role in the inflammatory process by adhering to and migrating under the endothelium, where they differentiate into macrophages. These macrophages can engulf and clear dead cells and tissue debris (15). Furthermore, the level of platelet-monocyte aggregates is considered an early marker of acute MI (16) and a new therapeutic target. Current analyses indicate that the extent of platelet-monocyte aggregation may directly translate to the intensity of myocardial inflammation. The interaction between platelets and monocytes has potential implications for the mid-and long-term processes of infarct healing and remodeling (17).

In this study, we identified three DEGs closely related to SA and MI: G protein subunit alpha q (GNAQ), elongator acetyltransferase complex subunit 3 (ELP3), and testin LIM domain protein (TES). First, GNAQ exhibited high expression in MI patients, while ELP3 and TES showed low expression. These genes are highly enriched in networks related to ribosome biogenesis, DNA replication, and innate immune deficiencies, suggesting they may influence MI by affecting these critical biological processes. Secondly, these DEGs are closely related to the abundance and function of immune cells. They influence the function of immune cells through different pathways, potentially playing a role in the occurrence and development of MI. Finally, these DEGs also show significant associations with immunopathological characteristics. GNAQ is positively correlated with some upregulated immunosuppressive genes, such as IL10RB, and negatively correlated with downregulated immunosuppressive genes. This indicates that GNAQ may influence the pathological process of MI by regulating immunosuppressive mechanisms. ELP3 and TES show the opposite correlation with these immunosuppressive genes, further supporting their crucial role in immune regulation.

To the best of our understanding, this research represents the first attempt to quantify the genetic causal relationship between SA and MI utilizing an MR framework. This MR study presents several significant advantages. Primarily, we eliminated genetic variations linked with potential confounding variables commonly identified in epidemiological studies, selecting only SNPs intimately associated with SA. Secondly, the substantial sample size of our MR investigation enhanced our statistical power, furnishing robust evidence for the existence of the relationship. Lastly, we implemented multiple sensitivity analyses to corroborate the durability of these findings. Notwithstanding the aforementioned advantages, certain limitations persist. Primarily, the employment of aggregate data from GWAS databases precluded our ability to evaluate the nonlinear association between SA and MI. Secondly, the predominant European ancestry of the study participants may minimize population stratification bias, yet it simultaneously constrains the generalizability of our results to diverse populations. Lastly, the decision to liberalize the *p*-value threshold to 1 × 10^–6^ for the inclusion of additional IVs (18,19), could potentially distort the depiction of the causal relationship with MI.

## Conclusion

In this research, we performed a univariate MR analysis to explore the causal link between SA and MI risk. Our findings indicated that SA is linked to a heightened risk of MI. Furthermore, we discovered three differentially expressed genes that play a role in the immune mechanisms driving MI pathogenesis. These genes offer new perspectives on the signaling pathways associated with SA and MI and could serve as novel targets for MI treatment and prevention.

## Data Accessibility Statement

The data analyzed in this study can be obtained from the FinnGen consortium database.

## Additional File

The additional file for this article can be found as follows:

10.5334/gh.1392.s1 Supplementary Material: Table S1. Genome-Wide Association Study summary data and expression quantitative trait loci studies’ data information.
