# Supplementary material for: Spontaneous Abortion and Myocardial Infarction: A Mendelian Randomization Investigation and Transcriptomic Analysis
Source: Glob Heart. 2025 Feb 6;20(1):12. doi: 10.5334/gh.1392 (PMC11804178; doi:10.5334/gh.1392)
Supplement: Supplementary Material: Table S1. — Genome-Wide Association Study summary data and expression quantitative trait loci studies’ data information. [file gh-20-1-1392-s1.pdf]

Table S1 Genome-Wide Association Study summary data and expression quantitative trait loci studies' data information.

GWAS ID	Traits	N of cases	N of controls	Sample size	Population	Description	Access address
R11_O15_ABORT_SPONTAN	Spontaneous abortion	20,775	180,063	200,838	European	Three or more consecutive spontaneous abortions.	https://console.cloud.google.com/storage/browser/_details/finngen-public-data-r11/summary_stats/finngen_R11_O15_ABORT_SPONTAN.gz;tab=live_object
R11_I9_MI_STRICT	MI	28,546	378,019	406,565	European	Coronary thrombosis: Coagulation of blood in any of the coronary vessels. The presence of a blood clot (thrombus) often leads to myocardial infarction.	https://console.cloud.google.com/storage/browser/_details/finngen-public-data-r11/summary_stats/finngen_R11_I9_MI_STRICT.gz;tab=live_object
